# Licochalcone A-Induced Apoptosis Through the Activation of p38MAPK Pathway Mediated Mitochondrial Pathways of Apoptosis in Human Osteosarcoma Cells In Vitro and In Vivo

**DOI:** 10.3390/cells8111441

**Published:** 2019-11-14

**Authors:** Renn-Chia Lin, Shun-Fa Yang, Hui-Ling Chiou, Shu-Ching Hsieh, Shiua-Hua Wen, Ko-Hsiu Lu, Yi-Hsien Hsieh

**Affiliations:** 1 Institute of Medicine, Chung Shan Medical University, Taichung 40201, Taiwanysf@csmu.edu.tw (S.-F.Y.); 2 Department of Orthopedics, Chung Shan Medical University Hospital, Chung Shan Medical University, Taichung 40201, Taiwan; 3 School of Medicine, Chung Shan Medical University, Taichung 40201, Taiwan; 4 School of Medical Laboratory and Biotechnology, Chung Shan Medical University, Taichung 40201, Taiwan; hlchiou@csmu.edu.tw; 5 Institute of Biochemistry, Microbiology, and Immunology, Chung Shan Medical University, Taichung 40201, Taiwan; s789580303705@yahoo.com.tw (S.-C.H.); leojuly30@hotmail.com (S.-H.W.); 6 Department of Medical Research, Chung Shan Medical University Hospital, Taichung 40201, Taiwan; 7 Clinical Laboratory, Chung Shan Medical University Hospital, Taichung 40201, Taiwan; 8 Department of Biochemistry, School of Medicine, Chung Shan Medical University, Taichung 40201, Taiwan

**Keywords:** LicA, osteosarcoma cells, MMPs, apoptosis, p38MAPK

## Abstract

Background: Licochalcone A (LicA) is isolated from the roots of *Glycyrrhiza glabra* and possesses antitumor and anti-invasive activities against several tumor cells. However, the antitumor effects of LicA on human osteosarcoma cells have yet to be demonstrated either in vitro or in vivo. Methods: Cell viability was measured by MTT assay. Apoptosis and mitochondrial dysfunction were detected with Annexin V/PI staining and JC-1 staining by flow cytometry. The expressions of caspase- or mitochondrial-related proteins were demonstrated by western blotting. Antitumor effect of LicA on 143B xenograft mice in vivo. Results: LicA could inhibit cell proliferation and induce apoptosis in human osteosarcoma cells, as evidenced by a decrease in cell viability, loss of mitochondrial membrane potentials, and activation of caspases. LicA treatment substantially reduced the expression of Bcl-2 and Mcl-1 and increased the expression of cleaved-caspase-3, cleaved-caspase-9, cleaved-PARP, and Bax in HOS and U2OS cells. Moreover, mitochondrial membrane potential and apoptosis suppression mediated by Z-VAD or tauroursodeoxycholic acid significantly reduced LicA-induced mitochondria-dependent apoptosis. The study also determined that LicA treatment induced p38MAPK phosphorylation, but siRNA-p38 or BIRB796 substantially reversed cell viability through the inhibition of mitochondria-dependent apoptosis pathways. Finally, an in vivo study revealed that LicA significantly inhibited 143B xenograft tumor growth. Conclusions: These findings demonstrate that LicA has antitumor activities against human osteosarcoma cells through p38MAPK regulation of mitochondria-mediated intrinsic apoptotic pathways in vitro and in vivo.

## 1. Introduction

Human osteosarcoma is a primary malignant bone tumor that occurs mainly in children and adolescents. Currently, the main conventional therapeutic treatment methods are chemotherapy and local surgery to control the primary lesions. However, the 5-year survival rate of patients with osteosarcoma treated with such methods is only approximately 60%; moreover, the methods do not completely increase patients’ survival rate [1], especially patients with osteosarcoma metastasis [2]. Therefore, developing new and effective anticancer drugs against osteosarcoma and increasing the survival of patients with osteosarcoma are imperative.

Evidence reveals that some natural products have demonstrated anticancer, anti-inflammatory, and apoptosis-inducing activities in various tumor cells; such compounds are also associated with low toxicity levels and few side effects [3]. Studies have demonstrated that *Glycyrrhiza glabra* is useful in the treatment of gastritis [4] and inflammation-related conditions [5]. Licochalcone A (LicA) is derived from the roots of *Glycyrrhiza glabra* [6]. Several studies have reported that it possesses antioxidant [7], anti-tumor growth [8], antimetastatic [9], and autophagy/apoptosis-inducing properties [10]. LicA inhibits lung cancer cell proliferation through endoplasmic reticulum (ER) stress activation [11]. It also induces cell cycle arrest of G2/M and ATM-Chk2 checkpoints in oral squamous cell carcinoma and osteosarcoma cancer cells, leading to cell apoptosis and autophagy [12,13]. The mitogen-activated protein kinase (MAPK) pathway was considered to be among the key mechanisms involved in tumor cell apoptosis, autophagy, and metastasis [14]. In addition, this pathway was considered to be involved in the proliferation and metastasis of osteosarcoma cancer cells [15]. The literature indicates that LicA inhibits the PI3K/AKT/mTOR pathway, which in turn leads to apoptosis and autophagy in breast cancer cells [16] and cervical cancer cells [17]. LicA-induced apoptosis occurs in nasopharyngeal carcinoma cells [18], head and neck squamous cell carcinoma [12] and oral cancer [19] through the activation of the p38MAPK and PI3K/AKT pathways. On the basis of the aforementioned reports and findings in the literature, LicA has potential antitumor and autophagy-inducing effects on various tumor cells; nevertheless, the molecular mechanism of LicA-induced mitochondria-dependent apoptosis in osteosarcoma cells remains unclear. Accordingly, the present study examined the antitumor effects and molecular mechanism of LicA against osteosarcoma cells in in vitro and in vivo xenograft osteosarcoma models.

## 2. Materials and Methods

### 2.1. Chemical Reagents and Antibody

LicA (BP0855) was purchased from Chengdu Biopurify Phytochemicals Ltd. (Chengdu, China). Primary antibodies against p-ERK, cleaved caspase-3, cleaved caspase-9, and cleaved poly (ADP-ribose) polymerase (PARP) were bought from Cell Signaling Technologies (Beverly, MA, USA). Primary antibodies against Bcl-2, Mcl-1, Bax, t-ERK, p-p38, t-p38, p-JNK, t-JNK, β-actin, and siRNA-p38 (sip38) were purchased from Santa Cruz Biotechnology (Santa Cruz, CA, USA). Moreover, 3-(4,5-dimethylthiazol-2-yl)-2,5-diphenyltetrazolium bromide (MTT) was purchased from Sigma-Aldrich (St. Louis, MO, USA). Z-VAD-FMK and tauroursodeoxycholic acid (TUDCA) were purchased from BioVision (Milpitas, CA, USA). BIRB 796 was bought from Tocris Bioscience (Minneapolis, MN, USA). Fetal bovine serum (FBS) was purchased from HyClone (Logan, UT, USA).

### 2.2. Cell Culture

Human ostecarcinoma HOS, U2OS, MG-63, and 143B cell lines were a gift from Dr. Shun-Fa Yang (Institute of Medicine, Chung Shan Medical University, Taichung, Taiwan). The normal osteoblast cell line MC3T3-E1 was gift from Dr. Chih-Hsin Tang (Department of Pharmacology, China Medical University, Taichung, Taiwan). The U2OS and MG-63 cells were maintained in Dulbecco’s Modified Eagle’s Medium (HyClone, UT, USA). The MC3T3-E1, HOS and 143B cells were cultured in MEM (HyClone, UT, USA) containing 10% FBS and 100 U/mL of penicillin-streptomycin (Invitrogen Life Technologies, Carlsbad, CA, USA) in a humidified incubator with 5% CO_2_ at 37 °C.

To examine the antitumor effects of LicA on osteosarcoma cells, various concentrations (0~100 μM) of LicA were added to these cells for 24 h. To inhibit the phosphorylation of p38MAPK expression or knock down p38 expression, 1 μM BIRB 796 was added to the cells for 2 h or sip38 (50 nM) was transfected onto the cells for 24 h before treatment with LicA (40 μM).

### 2.3. Cell Viability Assay

Cells (3 × 10^4^ cells/mL) were seeded in 24-well plates overnight at 37 °C. After 24 h of incubation, the cells were treated with LicA (0, 20, 40, 60, 80, and 100 μM) for 24 h to measure cell growth effects. The MTT (10 mg/mL) reagent was added, and the cells were incubated for 4 h. After the supernatant was removed, they were dissolved in isopropanol (500 μL/well). Subsequently, optical density was measured at 570 nm using a microplate reader (Bio-Rad Laboratories, Hercules, CA, USA). Cell viability is presented as a percentage of control cells

### 2.4. Annexin V/PI Staining by Flow Cytometry

An apoptosis assay was performed as described in a previous study [20]. After being treated with LicA at different concentrations for 24 h, the cells were harvested and analyzed using the Muse Annexin V and Dead Cell kit (Merck Millipore, Burlington, MA, USA) to determine cell apoptosis. The treated cells were collected and incubated with 5 μL of Annexin V-FITC and 5 μL of PI reagents at room temperature in a dark place for 15 min. The population of apoptotic cells was determined and analyzed using the Muse Cell Analyzer (Merck Millipore, Burlington, MA, USA).

### 2.5. Mitochondria Membrane Potential by Flow Cytometry

A mitochondria membrane potential was determined as a previous study [21]. Treatment with cells with different concentration of LicA for 24 h, and mitochondria membrane potential activity was assessed using a Muse MitoPotential kit (Merck Millipore, Burlington, MA, USA) and analyzed the data by Muse cell analyzer (EMD Millipore, Billerica, MA, USA).

### 2.6. siRNA Transfection Assay

The siRNA transfection assay was described in previous report [21]. U2OS cells were seeded to the 6 cm dish at about 75% of confluence for overnight. The si-p38 (50 nM) combined with Lipofectamine RNAiMAX Transfection Reagent (Thermo Fisher Scientific Inc, Waltham, MA, USA) and mixed for 20 min, then added to cultured U2OS cells for 48 h. Inhibition efficiency were detected by western blotting assay to confirm.

### 2.7. Western Blot Analysis

The U2OS and HOS cells were seeded in a 6 cm-dish and treated with various concentrations of LicA for 24 h. Subsequently, the cells were extracted with lysis buffer (200 μL), and quantitation was conducted using a Bradford protein analysis kit (Thermo Fisher Scientific Inc, Waltham, MA, USA). Total protein (25 μg) was separated by 10–12% SDS-PAGE and then transferred onto polyvinylidene difluoride membranes (Merck Millipore, Burlington, MA, USA); the membranes were blocked with 5% nonfat dry milk in Tris-buffered saline with Tween-20 buffer. The membranes were hybridized with antibodies against cleaved caspase-3 (1:1000), cleaved caspase-9 (1:1000), cleaved PARP (1:1000), Bcl-2 (1:1000), Mcl-1 (1:1000), Bax (1:1000), p-ERK (1:2000), t-ERK (1:1000), p-p38 (1:1000), t-p38 (1:1000), p-JNK (1:1000), t-JNK (1:1000), and β-actin (1:5000). The membranes were incubated with a peroxidase-conjugated secondary antibody (1:10000) for 1 h. Finally, the blot membranes were detected using chemiluminescent signals and quantitated using a Luminescent Image Analyzer (LAS 4000 mini, GE Healthcare Bio-Sciences, Pittsburgh, PA, USA).

### 2.8. In Vivo Nude Mice Assay and Safety Evaluation

BALB/c mice (4–5 weeks old) were purchased from the National Laboratory Animal Center (Taipei, Taiwan) and all animal experiment were handled according to approval of the Animal Care and Use Committee at Chung Shan Medical University (IACUC: 2271). The 143B cells (1 × 10^6^/100 μL) were injected into the right flank of the nude mice subcutaneously. After 1 weeks and the tumor raised up approximately 85 mm^3^, the animals were orally administered DMSO (n = 5) or LicA (10 mg/kg; n = 5) twice once a week for 5 weeks. The tumor size of the mice in each group was measured every 7 days. Tumor volume was determined using the following formula: 1/2 (L1 × L2 × H), where L1 represents the long diameter, L2 represents the short diameter, and H represents the height of the tumor. After treatment for 5 weeks, the mice were sacrificed and tumor was removed. The tumor weight of the mice in each group was measured. Liver, heart, renal spleen, and lung samples were removed for safety evaluation and hematoxylin and eosin (HE) staining.

### 2.9. Statistical Analysis

Statistical analysis was performed using the SPSS 12.0 and GraphPad Prism 5.0 software packages. Data are expressed as mean ± standard deviation. One-way analysis of variance or Student’s unpaired t test was conducted using SPSS 12.0 to establish the differences between two values. A *p* value of <0.05 was considered to be statistically significant.

## 3. Results

### 3.1. Effect of LicA on the Growth of Human Osteosarcoma Cells and Normal Osteoblast Cells

**Figure 1:**
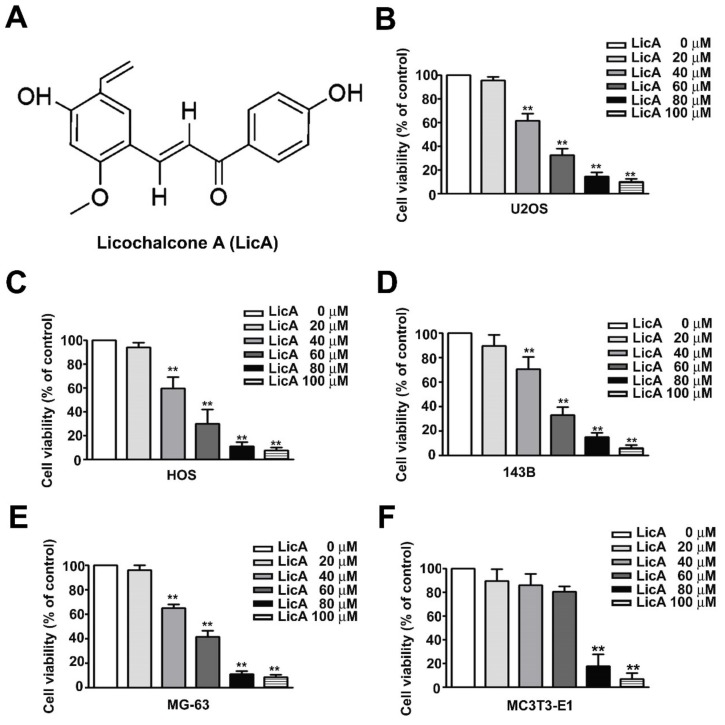
Effect of LicA on cell viability of osteosarcoma cells and normal osteoblast cells. (**A**) Chemical structure of LicA. (**B**–**E**) Human osteosarcoma cell lines (U2OS, HOS, 143B, and MG-63) and (**F**) normal osteoblast cell (MC3T3-E1) were treated with various concentrations of LicA (0, 20, 40, 60, 80, or 100 μM) for 24 h. Cell viability was determined by the MTT assay. Data are presented as mean ± standard error for three independent experiments. ** *p* < 0.01 compared with controls.

The chemical structure of Licochalcone A (LicA) as shown in Figure 1A. To examine the effects of LicA on cell viability, osteosarcoma cell lines (U2OS, HOS, 143B, MG-63) and normal osteoblast cell (MC3T3-E1) were treated with increased concentrations of LicA for 24 h, and cell viability was assessed through the MTT assay. The results showed that LicA significantly reduced cell viability in a dose-dependent manner (Figure 1B–E). As shown in Figure 1F, a little toxicity of cell viability in normal osteoblast cell (MC3T3-E1) was observed in treated with high dose of LicA (60 µM). Therefore, treated the concentrations (20, 40, and 60 μM) of LicA were chosen for the further in vitro cell experiment.

### 3.2. Effect of LicA on Cell Apoptosis of Human Osteosarcoma Cell

**Figure 2:**
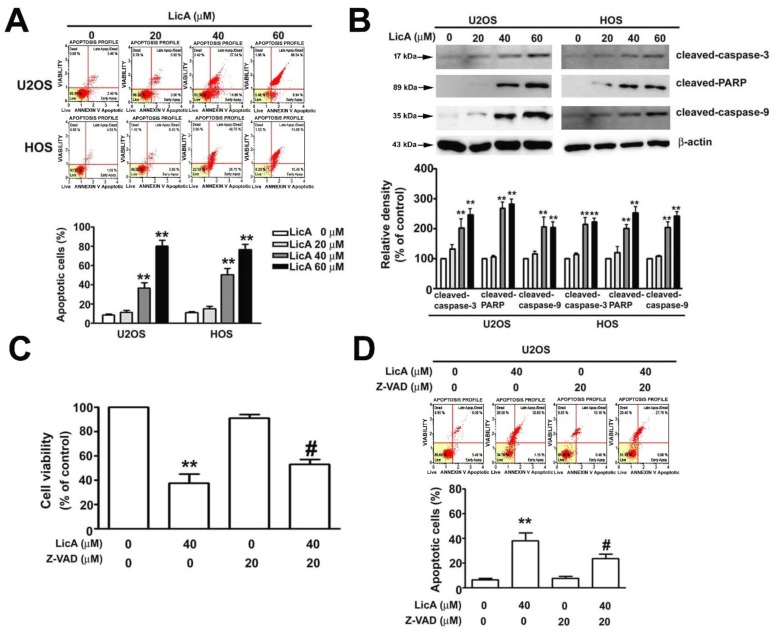
LicA induces apoptosis in osteosarcoma cells. U2OS and HOS were treated with various concentrations of LicA (0, 20, 40, or 60 μM) for 24 h. (**A**) Apoptotic profile was assessed using the Muse Annexin V and Dead Cell Assay by flow cytometry. Quantitative results of apoptotic cells (Annexin V–stained cells) are presented in the bottom plot. (**B**) Protein expression levels of cleaved caspase-3, cleaved PARP, and cleaved caspase-9 were assessed through western blot ting. β-actin was employed as an internal control. Relative quantitative results are depicted in the bottom plot. Cells were pretreated with or without 20 μM Z-VAD for 2 h and further treated with or without 40 μM LicA for 24 h. (**C**) Cell viability was determined by the MTT assay. (**D**) Apoptotic profile was detected though the Muse Annexin V and Dead Cell Assay by flow cytometry. Quantitative results of apoptotic cells (Annexin V–stained cells) are presented in the bottom plot. ** *p* < 0.01 compared with controls. # *p* < 0.01 compared with LicA treatment alone.

To determine whether LicA inhibits osteosarcoma cell viability by inducting cell apoptosis, the U2OS and HOS cells were treated with various LicA concentrations were detected with Annexin V and dead cell assay by flow cytometry for 24 h, which resulted in a dose-dependent increase in the percentage of apoptotic cells (Figure 2A). The results also indicated an apoptotic response that involved an increase in cleaved caspase-3, cleaved caspase-9, and cleaved PARP (Figure 2B). In addition, the U2OS and HOS cells were pretreated with a pancaspase inhibitor, Z-VAD, followed by 24 h of incubation with LicA. Z-VAD partially attenuated the growth inhibition (Figure 2C) and apoptosis induction (Figure 2D) by LicA. These results indicate that LicA inhibits the growth of osteosarcoma cells through activation of caspase-dependent apoptosis.

### 3.3. Effect of LicA on Mitochondrial Membrane Potential in Human Osteosarcoma Cells

**Figure 3:**
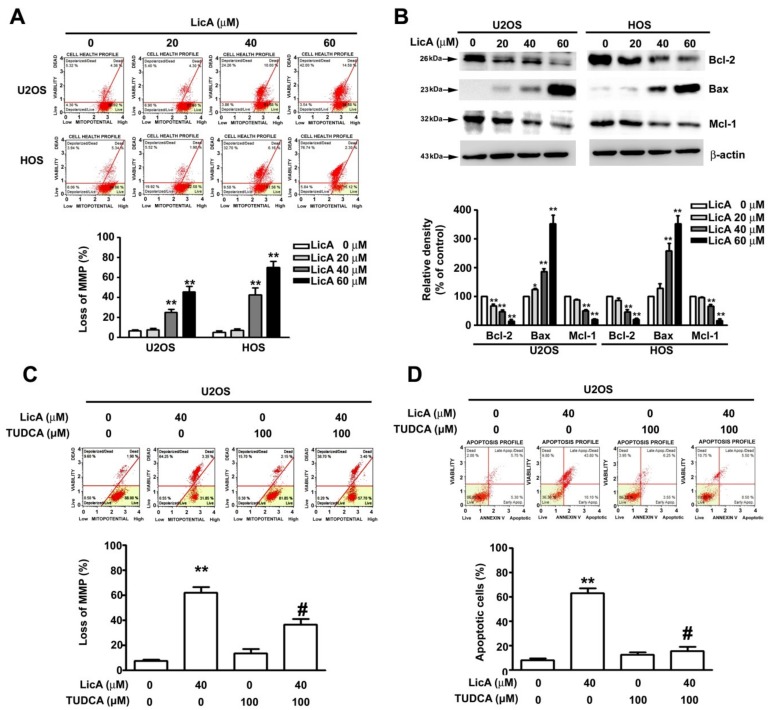
LicA induces mitochondrial dysfunction mediated apoptosis in osteosarcoma cells. U2OS and HOS were treated with various concentrations of LicA (0, 20, 40, or 60 μM) for 24 h. (**A**) Alterations in mitochondrial membrane potential were measured through the Muse Mitopotential assay by flow cytometry. Quantitative results of depolarized cells are depicted in the bottom plot. (**B**) Protein expression levels of Bcl-2, Bax, and Mcl-1 were assessed through western blot ting. β-actin was employed as an internal control. Relative quantitative results are shown in the bottom plot. (**C**) Cells were pretreated with or without 100 μM tauroursodeoxycholic acid for 2 h and further treated with or without 40 μM LicA for 24 h. Alterations in MMP were measured through the Muse Mitopotential assay by flow cytometry. (**D**) Apoptotic profile was assessed using the Muse Annexin V and Dead Cell Assay by flow cytometry. Quantitative results are presented in the bottom plot. * *p* < 0.05; ** *p* < 0.01 compared with controls. # *p* < 0.01 compared with LicA treatment alone.

To investigate the underlying mechanism of LicA-induced apoptosis, the U2OS and HOS cells were treated with various concentrations of LicA for 24 h and subjected to the Muse Mitopotential assay. As illustrated in Figure 3A, LicA significantly increased the portion of depolarized cells. Furthermore, LicA treatment resulted in the up-regulation of proapoptotic Bax proteins and the down-regulation of antiapoptotic Bcl-2 and Mcl-1 proteins (Figure 3B). The addition of the mitochondrial apoptosis inhibitor TUDCA also significantly reduced the loss of membrane potential (Figure 3C) and apoptosis (Figure 3D) induced by LicA in the U2OS cells.

### 3.4. LicA Activates p38MAPK in Human Osteosarcoma Cells

**Figure 4:**
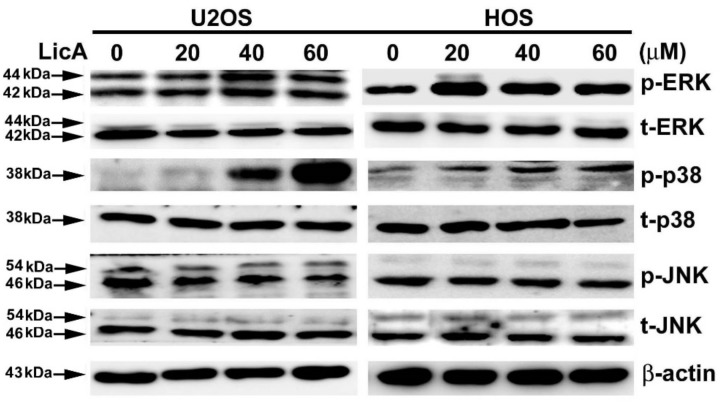
LicA activates the p38 mitogen-activated protein kinase pathway in osteosarcoma cells. U2OS and HOS were treated with various concentrations of LicA (0, 20, 40, or 60 μM) for 24 h. Cells were then harvested for western blotting to observe the activation of the MAPK signaling pathway. β-actin was employed as internal control.

The MAPK signaling pathway regulates apoptosis pathways [22]. To assess whether LicA-induced apoptosis was mediated by the MAPK signaling pathway, the U2OS and HOS cells were treated with different concentrations of LicA for 24 h and analyzed using western blotting. The results revealed that LicA treatment dose-dependently phosphorylated p38MAPK only, and did not phosphorylate ERK and JNK (Figure 4).

### 3.5. Activation of p38MAPK Involved in LicA Induces Apoptosis in Human Osteosarcoma Cells

**Figure 5:**
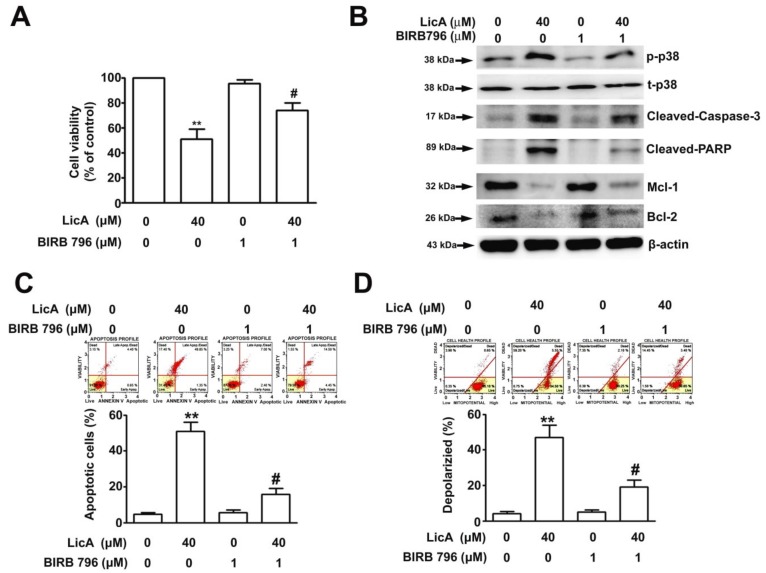
BIRB 796 attenuates LicA-induced apoptosis in osteosarcoma cells. U2OS cells were pretreated with or without BIRB796 (1 μM) for 2 h and further treated with or without 40 μM LicA for 24 h. (**A**) Cell viability was determined through the MTT assay. (**B**) Protein expression levels of p-p38, t-p38, cleaved caspase-3, cleaved-PARP, Mcl-1, and Bcl-2 were assessed through western blotting. β-actin was employed as an internal control. (**C**) Apoptotic profile was assessed by the Muse Annexin V and Dead Cell Assay by flow cytometry. (**D**) Alterations in MMP were measured through the Muse Mitopotential assay by flow cytometry. Quantitative results are presented in the bottom plot. ** *p* < 0.01 compared with controls. # *p* < 0.01 compared with LicA treatment alone.

**Figure 6:**
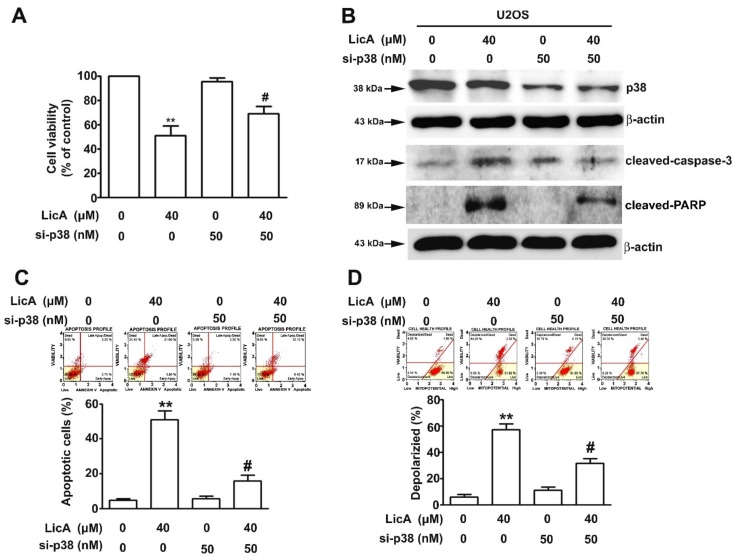
RNA interference of p38 attenuates LicA-induced apoptosis in osteosarcoma cells. U2OS cells were treated with or without 40 μM LicA in the presence or absence of si-p38 (50 nM) for 48 h. (**A**) Cell viability was determined using the MTT assay. (**B**) Protein expression levels of p38, cleaved caspase-3, and cleaved PARP were assessed through western blot ting. β-actin was employed as an internal control. (**C**) Apoptotic profile was assessed through the Muse Annexin V and Dead Cell Assay by flow cytometry. (**D**) Alterations in MMP were measured through the Muse Mitopotential assay by flow cytometry. Quantitative results are depicted in the bottom plot. ** *p* < 0.01 compared with controls. # *p* < 0.01 compared with LicA treatment alone.

To understand the role of the p38 signaling pathway in LicA-induced apoptosis, the U2OS cells were pretreated with BIRB796, a specific p38 inhibitor. BIRB796 significantly ameliorated the LicA-induced growth inhibition (Figure 5A). The western blot analysis results indicated BIRB796 treatment decrease in p38 phosphorylation, apoptotic protein responses (cleaved caspase-3 and cleaved PARP), and an increase in antiapoptotic proteins (Mcl-1 and Bcl-2) by LicA treatment, compared with LicA alone (Figure 5B). The Muse Annexin V and Mitopotential assays were further employed to assess the effect of BIRB796 on LicA-induced apoptosis. As revealed in Figure 5C,D, BIRB796 treatment significantly decreased both the apoptosis and depolarized portion of the LicA-treated cells. To further address the importance of p38 signaling pathway in LicA-induced apoptosis, RNA interference of p38 was analyzed in the U2OS cells. Similar to BIRB796, si-p38 significantly attenuated the LicA-induced growth inhibition (Figure 6A), apoptotic responses (Figure 6B), and percentage of apoptotic and depolarized cells (Figure 6C,D). Taken together, these results demonstrate that LicA inhibits the growth of osteosarcoma cells through a p38-mediated intrinsic apoptotic pathway.

### 3.6. LicA Suppresses the Growth of 143B Xenografts In Vivo and During Safety Evaluation

**Figure 7:**
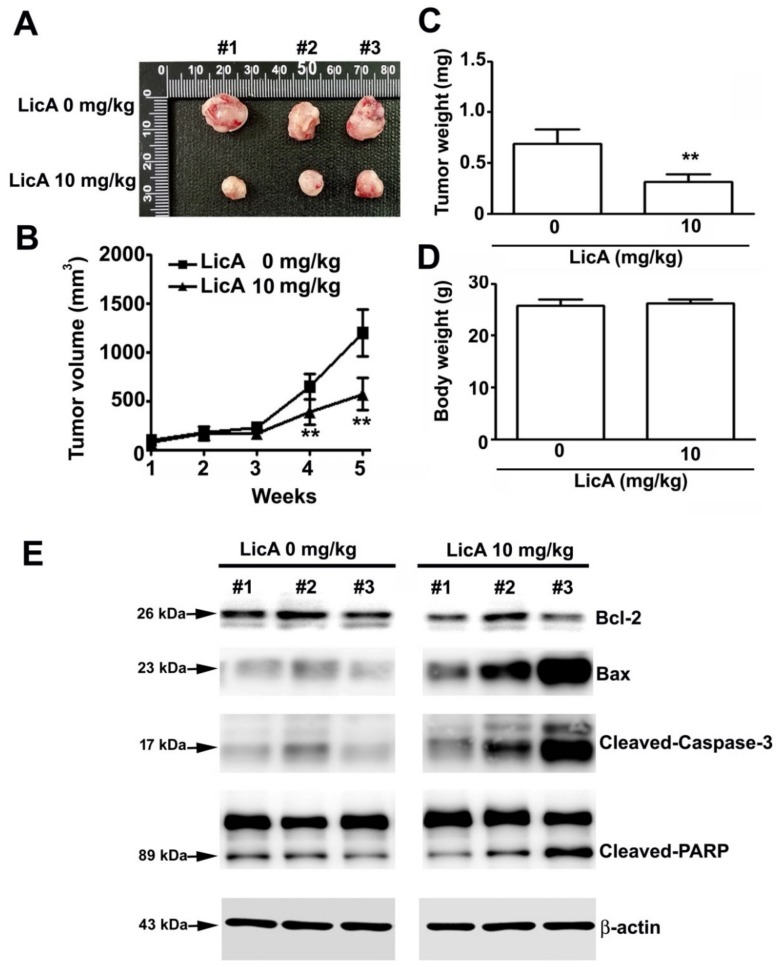
LicA suppresses the growth of 143B xenografts in vivo. BALB/c nude mice were subcutaneously inoculated with 143B cells. After a tumor establishment period (1 week), LicA (10 mg/kg, twice per week) or DMSO was orally administered to the nude mice. (**A**) Representative image of the tumors. (**B**) Average tumor volume and (**C**) average tumor weight. (**D**) Average body weight of the mice. (**E**) Tumor tissues were harvested and subjected to western blot ting to examine the expression level of Bcl-2, Bax, cleaved-caspase-3, and cleaved-PARP. β-actin was employed as an internal control. ** *p* < 0.01 compared with controls.

**Figure 8:**
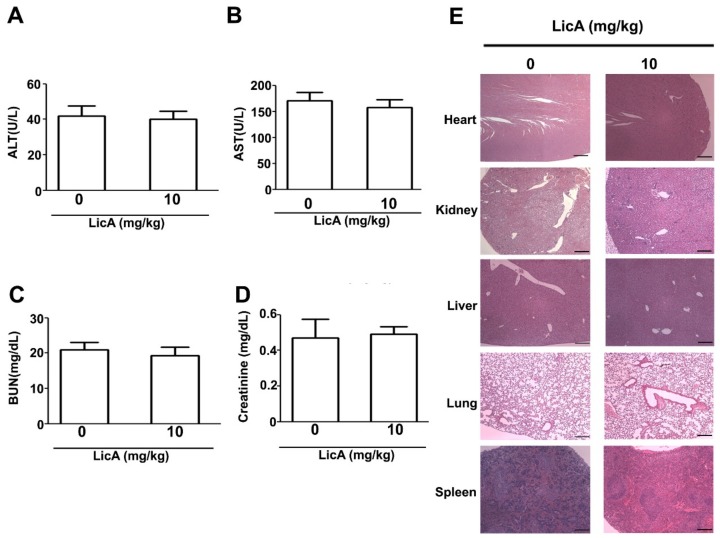
In vivo safety evaluation of LicA. After the designated treatment period, tumor-inoculated mice were sacrificed. The blood and major organs of the mice were collected to evaluate the in vivo toxicity of LicA. (**A**–**D**) Serum concentrations of AST, ALT, BUN, and creatinine from both groups were measured. (**E**). Histopathological alteration in the major organs (heart, liver, spleen, lung, and kidney) was assessed after HE staining.

Finally, to investigate the effect of LicA on the in vivo growth of osteosarcoma cells, the 143B cells were subcutaneously inoculated onto nude BALB/c mice. As illustrated in Figure 7A, oral administration of LicA significantly inhibited tumor growth (Figure 7B) and tumor weight (Figure 7C), whereas the body weights of treated and untreated mice remained equal (Figure 7D). After the sacrifice of the mice, the tumor xenografts were harvested and subjected to western blot analysis, which revealed an increased apoptotic response (Bax, cleaved-caspase-9 and cleaved-PARP) and the downregulation of proapoptotic proteins (Bcl-2) (Figure 7E), similar to the in vitro analysis results. Moreover, blood biochemical analyses indicated no difference in serum AST and ALT levels, suggesting that there was no liver toxicity (Figure 8A,B). Similarly, the serum BUN and creatinine levels did not differ, indicating that LicA treatment did not cause kidney damage or nephrotoxicity (Figure 8C,D). Furthermore, the major organs (heart, lung, liver, kidney, and spleen) of the sacrificed mice were subjected to HE staining for histopathological examination. As shown in Figure 8E, LicA did not induce any apparent damage to these organs. In conclusion, these results indicate that LicA administration can inhibit the growth of 143B xenograft tumors while remaining safe to mice.

## 4. Discussion

**Figure 9:**
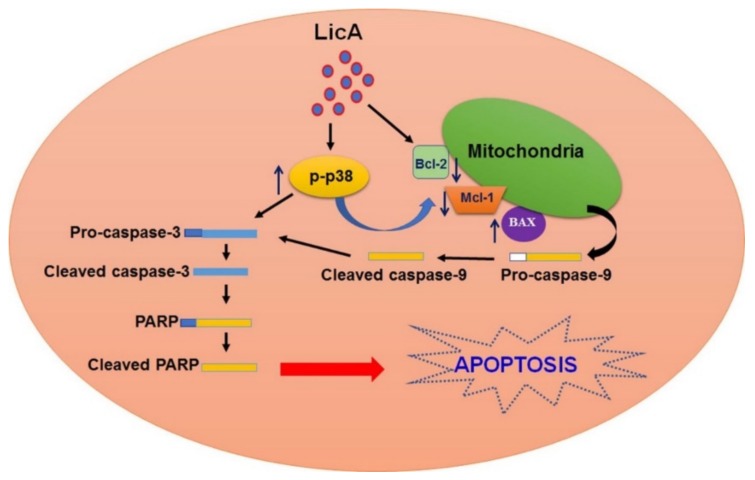
A predicted model for LicA-induce mitochondrial mediated apoptosis in human osteosarcoma cells. LicA has antitumor activities against human osteosarcoma cells dependent on mitochondria-mediated intrinsic apoptotic pathways, which results in activating p38MAPK pathways.

LicA is a natural product and the main active compound in the roots of licorice. Recent research has extensively documented the anticancer and antimetastatic activities of LicA in various tumor cells [10,23,24], nevertheless, little is known about the effects of LicA on human osteosarcoma cells. In this study, we used in vitro and in vivo models to examine whether LicA inhibits the cell viability and induces mitochondrial apoptosis in osteosarcoma cells (U2OS, HOS, MG63, and 143B) through the activation of the p38MAPK pathway (Figure 9). The study results provide new evidence supporting the development of LicA against osteosarcoma cells.

Recent reports have suggested that inducing tumor cell apoptosis is another effective and key strategy for the treatment of cancer. An apoptotic signaling pathway can be divided into intrinsic and extrinsic pathways, and LicA was reported to induce intracellular reactive oxygen species (ROS) generation, cell cycle arrest, and intrinsic and extrinsic pathway activation in human hepatocellular carcinoma cells [25]. LicA induced apoptosis is also associated with mitochondrial dysfunction, intracellular Ca^2+^ release, and ER stress in human bladder cancer cells [26]. Previous reports have demonstrated that LicA induces apoptosis in various tumor cells, including non-small cell lung cancer cells [10], breast cancer cells [16], and malignant pleural mesothelioma [27], through the activation of mitochondria-related apoptotic or autophagic apoptotic pathways. In addition, another major factor in apoptosis progression is mitochondrial function. Mitochondria not only regulate cell growth and metabolism but also produce energy [28]. The antiapoptotic proteins Bcl-2 and Mcl-1 prevent Bax and Bak homo-oligomerization, which leads to the inhibition of apoptosis [29]. According to these results, the inhibition of mitochondrial dysfunction by pretreatment with TUDCA (mitochondrial apoptosis inhibitor) could reverse LicA-inhibited cell viability. Our results demonstrate that LicA induced osteosarcoma cell mitochondrial dysfunction through a decrease in the expression of Bcl-2 and Mcl-1 and an increase in the expression of Bax.

The role of p38MAPK in cell proliferation and apoptosis has been extensively studied [30]. Accumulating bodies of evidence suggest that the role of p38MAPK is controversial in several tumor cells. This variation may be associated with the applied stimuli and duration as well as the specific characteristics and types of cells. In vitro and in vivo research demonstrated that hydroxysafflor yellow A induces the apoptosis of HepG2 cells by substantially inhibiting the phosphorylation of the p38MAPK pathway [31]. Other evidence reveals that NK007 induces G1/S arrest through the activation of phosphorylated p38MAPK expression and degradation of HK2 expression associated with acidification and oxygen consumption rates [32]. In our previous study, α-mangostin enhanced ROS generation, mitochondrial dysfunction, and apoptosis through the activation of the ASK1/p38 signaling pathway in cervical cancer cells [33]. However, other studies have demonstrated that the activation of p38MAPK activity was dependent on apoptosis. Zhang et al. reported that a combination of dihydroartemisinin with carboplatin induced cell cycle arrest and apoptosis in Lewis lung carcinoma cells through the activation of p38MAPK [34]. Albumin induces ER stress depending on apoptosis through the activation of the ER-mediated p38MAPK/caspase 12 pathway in podocyte apoptosis [35]. Other studies have shown that fruit EGCG and polyphenol induced apoptosis through mitochondrial pathways and the modulation of p38MAPK activity in human breast cancer cells [36] and colon cancer cells [37]. Similarly, our study suggested that LicA increased p-p38MAPK expression; BIRB 897 significantly reversed cell viability, mitochondrial membrane potential (MMP), and caspase expression through LicA treatment; and siRNA-p38 transfection exhibited the same effect. Therefore, additional studies are required to examine the molecular mechanisms through which LicA-regulated mitochondria-related proapoptotic proteins, including Bax and Bcl-2, or caspase-3/-9 proteins activate protein expression pathways that control the interplay between p38MAPK and apoptosis induction.

Overall, our results indicate that LicA significantly induced mitochondrial apoptosis in human osteosarcoma cells in vitro and in vivo, as expressed by the increase in cleaved caspase-3, -9, and PARP protein expression and Bax/Bcl-2 ratio. Notably, the increase in p38MAPK-mediated activation of caspases or mitochondria-related protein expression can promote the mitochondrial apoptosis of human osteosarcoma cells. These results provide sufficient in vitro and in vivo evidence to demonstrate the anticancer effects of LicA. LicA may be a useful and effective therapeutic strategy against human osteosarcoma cells in the future
